# Prevalence of Obesity and the Relationship between the Body Mass Index and Body Fat: Cross-Sectional, Population-Based Data

**DOI:** 10.1371/journal.pone.0029580

**Published:** 2012-01-13

**Authors:** Julie A. Pasco, Geoffrey C. Nicholson, Sharon L. Brennan, Mark A. Kotowicz

**Affiliations:** 1 School of Medicine, Deakin University, Geelong, Victoria, Australia; 2 NorthWest Academic Centre, Department of Medicine, The University of Melbourne, St Albans, Victoria, Australia; 3 Rural Clinical School, School of Medicine, The University of Queensland, Toowoomba, Queensland, Australia; Johns Hopkins Bloomberg School of Public Health, United States of America

## Abstract

**Background:**

Anthropometric measures such as the body mass index (BMI) and waist circumference are widely used as convenient indices of adiposity, yet there are limitations in their estimates of body fat. We aimed to determine the prevalence of obesity using criteria based on the BMI and waist circumference, and to examine the relationship between the BMI and body fat.

**Methodology/Principal Findings:**

This population-based, cross-sectional study was conducted as part of the Geelong Osteoporosis Study. A random sample of 1,467 men and 1,076 women aged 20–96 years was assessed 2001–2008. Overweight and obesity were identified according to BMI (overweight 25.0–29.9 kg/m^2^; obesity ≥30.0 kg/m^2^) and waist circumference (overweight men 94.0–101.9 cm; women 80.0–87.9 cm; obesity men ≥102.0 cm, women ≥88.0 cm); body fat mass was assessed using dual energy X-ray absorptiometry; height and weight were measured and lifestyle factors documented by self-report. According to the BMI, 45.1% (95%CI 42.4–47.9) of men and 30.2% (95%CI 27.4–33.0) of women were overweight and a further 20.2% (95%CI 18.0–22.4) of men and 28.6% (95%CI 25.8–31.3) of women were obese. Using waist circumference, 27.5% (95%CI 25.1–30.0) of men and 23.3% (95%CI 20.8–25.9) of women were overweight, and 29.3% (95%CI 26.9–31.7) of men and 44.1% (95%CI 41.2–47.1) of women, obese. Both criteria indicate that approximately 60% of the population exceeded recommended thresholds for healthy body habitus. There was no consistent pattern apparent between BMI and energy intake. Compared with women, BMI overestimated adiposity in men, whose excess weight was largely attributable to muscular body builds and greater bone mass. BMI also underestimated adiposity in the elderly. Regression models including gender, age and BMI explained 0.825 of the variance in percent body fat.

**Conclusions/Significance:**

As the BMI does not account for differences in body composition, we suggest that gender- and age-specific thresholds should be considered when the BMI is used to indicate adiposity.

## Introduction

In the nineteenth century, the mathematician and social statistician, Adolphe Quetelet, observed that the weight of the average man was proportional to the square of the height [1]. The ratio of body weight measured in kilograms divided by the square of the height measured in metres was termed the Quetelet's Index and later renamed the Body Mass Index (BMI) [2]. Had Quetelet based his studies on human growth in the developed world during the 21^st^ century, the constancy of the ratio may have been obscured by the widespread prevalence of obesity. As in the US [3] and Great Britain [4], there has been a marked rise in the prevalence of obesity in Australia [5], particularly since the 1980s. BMI data from the nationwide AusDiab study conducted 1999–2000 indicated that the prevalence of obesity in urban Australia had risen 2.5-fold since the 1980 National Heart Foundation of Australia survey [5]. This alarming rise is of public health concern, because obesity is associated with an increased risk for developing hypertension, lipid disorders, type 2 diabetes, heart disease [6], stroke [7], osteoarthritis [8] and certain cancers [9]. The cost of obesity to the Australian health system exceeded $8 billion in 2008, and this included costs associated with consequent metabolic disease, cardiac disease and surgical complications [10]. Furthermore, obesity is associated with a modestly increased risk for early all-cause mortality [11]. It seems likely that health advantages inspired by modern medicine are being eroded by the current obesity epidemic.

Although the BMI is widely utilised as an anthropometric estimate of general adiposity, the failure to identify differences in body composition and body fat distribution limit its usefulness. In recognition that visceral fat accumulation increases the risk for metabolic disease, waist circumference was promoted as an alternative surrogate measure of obesity [12] and ratios such as waist-to-hip [13], [14] and waist-to-height [15] have also been investigated as markers of risk for metabolic disease. However, few studies have reported population characteristics based on waist circumference.

In this cross-sectional study of men and women enrolled in the Geelong Osteoporosis Study (GOS), we aimed to describe the prevalence of overweight and obesity in Australian adults according to current criteria based on the BMI and waist circumference. Furthermore, we have investigated the relationship between the BMI and body fat determined by dual energy x-ray absorptiometry (DXA).

## Methods

### Ethics statement

The Barwon Health Human Research Ethics Committee approved the study. All participants gave written, informed consent.

### Subjects

The GOS is a population-based study of adult men and women aged 20 years and over, randomly-selected from the Commonwealth electoral rolls for the Barwon Statistical Division in south-eastern Australia. This region is ideal for epidemiological research because the population is large enough (259,013 total population; 91,078 men and 98,740 women aged 20 years and over) and suitably diverse to be representative of the nation [16]. Baseline assessments for the GOS commenced in 1993 for women (1,494 recruited, 77% response) and in 2001 for men (1,467 recruited, 67% response). This analysis utilises data collected at the 10-year follow-up assessment for women (882 of the eligible women were assessed at the 10-year follow-up with 82% response, 2003–2008) and baseline assessment for men (2001–2006) [16]. A further sample of 194 women aged 20–29 yr was also randomly generated (2005–2008, 82% response) and incorporated into the female cohort. Thus, data from 1,467 men and 1,076 women were included in analyses for determining prevalence of overweight and obesity, and for assessing the associations between BMI, waist circumference and lifestyle; almost all of the cohort (99%) was white.

A sub-group of 1,299 (89%) men and 855 (79%) women had whole body scans using DXA that provided valid data on body fat mass; 167 men and 221 women were excluded from statistical modelling for predicting fat mass and percentage body fat (%BF) from BMI because they were unable to assume the correct position for accurate scanning (n = 189; 34 of these individuals weighed 120 kg or more, which exceeded the scanner upper load), had prostheses or implants such as pacemakers, stents or breast augmentation (n = 135) or jewellery that could not be removed (n = 13), or they did not have a whole body DXA scan (n = 52).

### Measures

Standing height without shoes was measured to the nearest 0.1 cm using a wall-mounted stadiometer; weight was measured to the nearest 0.1 kg using electronic scales; waist (smallest circumference between the lower rib and iliac crest) and hip (maximal gluteal) circumferences were measured in a horizontal plane with a narrow, non-elastic tape measure [17]. Subjects were categorised as underweight if BMI <18.5 kg/m^2^, overweight if BMI was in the range 25.0–29.9 kg/m^2^, and obese if BMI ≥30.0 kg/m^2^ [12]. Obesity was also grouped as grade I if BMI 30.0–34.9 kg/m^2^, grade II if BMI 35.0–39.9 kg/m^2^, and grade III if BMI ≥40.0 kg/m^2^. Waist circumferences of 94.0–101.9 and ≥102.0 cm for men, and 80.0–87.9 and ≥88.0 cm for women, were used to classify overweight and obesity, respectively [12]. Whole body scans were performed using DXA (Lunar DPX-L or Prodigy Pro), which provided estimates of body fat mass, ‘lean’ mass (comprising muscle, skin, connective tissue and the lean component of adipose tissue - water and protein [18]) and bone mineral content (BMC). The %BF was calculated as body fat mass expressed as a percentage of the whole body mass from DXA (sum of body fat mass, lean mass and BMC). All clinical measures were performed by trained personnel.

Dietary intakes, including total energy intake (EI) from food and alcohol, were estimated using a food frequency questionnaire developed by the Cancer Council of Victoria [19] and validated for assessing dietary intakes in the Australian population [20]. The questionnaire recorded the individual's habitual consumption of 74 foods and six alcoholic beverages over the preceding 12-month period on a 10-point frequency scale. These responses were supplemented with information about portion sizes and food types. Mean daily alcohol and energy intakes were computed from the dietary data by means of the nutrient composition tables in the NUTTAB95 database (Food Standards Australia New Zealand, Canberra, 1995). Basal metabolic rate (BMR) was predicted from age, weight and gender [21] and EI/BMR calculated with both estimates expressed in megajoules; individuals with low ratios were identified as EI/BMR <0.9.

### Statistics

Waist measurements were not obtained for 21 men and 22 women; values were computed using prediction equations based on gender, age and weight and included in prevalence estimates for overweight and obesity based on waist circumference. To determine the prevalence of obesity according to BMI, age-stratified samples of men and women were standardised to national age-profiles (Australian Bureau of Statistics, Cat. No. 2068.0 – 2006 Census Tables). Gender differences in prevalence of obesity based on BMI and waist circumference were determined using age-stratified data; tests of homogeneity across age strata were performed in developing these models. For direct comparison with the AusDiab study, prevalence data for obesity were standardised to the 1998 Australian population aged ≥25 years (Australian Bureau of Statistics, Cat. No. 3201.0 – June 1997 to June 1998).

Multiple regression techniques were used to determine gender differences in the linear relationships between BMI and body fat mass and second order polynomial relationship between BMI and %BF. Polynomial relationships were centred about the mean to reduce collinearity. In validating the models, interaction terms between exposure variables were considered, to identify effect modifiers; interaction terms were retained in the models if p<0.05. Prediction equations for %BF from BMI were developed taking into account the effects of gender and age, and subsequently lean mass and BMC. Statistical analyses were performed using Stata (release 9.0 Statacorp, College Station, TX, USA) and Minitab (version 15; Minitab, State College, PA, USA).

## Results

### BMI for determining prevalence of obesity

Our data indicate that, according to BMI criteria, 1.0% of individuals were underweight. Our data also indicate that 37.5% of individuals were overweight and a further 24.5% were obese; prevalence figures for each gender are listed in Table 1. The prevalence of obesity according to BMI was lower for men than for women (RR = 0.71, 95% CI 0.62–0.81). Pooled overweight and obesity data show that 65.3% men and 58.8% women had BMI values above the recommended threshold (62.0% overall). The prevalence of obesity defined by BMI and age-standardised to the 1998 Australian population for ages ≥25 years has risen from 20.8% (95%CI 18.4–23.1) in 1999–2000 [5] to 25.3% (95%CI 23.4–27.2) in 2001–2008.

**Table 1:** Age-standardised prevalence (mean percentage and 95% CI) of overweight and obesity determined by BMI and waist circumference.

Measurement	Classification	Men	Women
BMI*	Overweight	45.1 (42.4–47.9)	30.2 (27.4–33.0)
	Obese	20.2 (18.0–22.4)	28.6 (25.8–31.3)
	Obesity grade I	16.0 (15.4–16.7)	17.0 (14.7–19.5)
	Obesity grade II	3.1 (2.2–4.1)	7.4 (5.8–9.0)
	Obesity grade III	1.0 (0.5–1.6)	4.3 (3.0–5.6)
Waist circumference¥	Overweight	27.5 (25.1–30.0)	23.3 (20.8–25.9)
	Obese	29.3 (26.9–31.7)	44.1 (41.2–47.1)

CI = confidence interval; BMI = body mass index.

*BMI: Overweight 25.0–29.9 kg/m^2^; obese ≥30.0 kg/m^2^; obesity grade I 30.0–34.9 kg/m^2^; grade II 35.0–39.9 kg/m^2^; grade III ≥40 kg/m^2^.

¥ Waist circumference overweight 94.0–101.9 cm for men and 80.0–87.9 cm for women; obesity ≥102.0 cm for men and ≥88.0 cm for women.

BMI was correlated to other indices of adiposity in both men and women; Pearson's correlation was 0.88 for weight, 0.88 for waist, 0.49 for waist/hip ratio and 0.85 for waist/height in men, and 0.92 for weight, 0.87 for waist, 0.32 for waist/hip ratio and 0.86 for waist/height in women (all p<0.001). Gender-specific characteristics of the study population are presented in Tables 2 and 3, for the whole group and according to categories of BMI; because of small numbers, underweight individuals were included in the category BMI <25.0 kg/m^2^ and obesity grades I, II and III were pooled to form the BMI ≥30 kg/m^2^ category. Men and women with BMI <25.0 kg/m^2^ were younger and taller than those who were overweight and obese. The waist/hip and waist/height ratios both increased across increasing categories of BMI. Waist/hip ratio ≥0.80 was present in all but nine men and in 798 (74.2%) of women.

**Table 2:** Characteristics of men, overall and according to BMI categories; data presented as mean (±SD), median (IQR) or frequency (%).

	All	BMI category			
		<25.0	25.0–29.9	≥30	p
	n = 1467	n = 476	n = 687	n = 304	
Age (yr)	56 (39–73)	48 (30–73)	58 (44–74)	59 (44–71)	<0.001
Weight (kg)	82.7 (±14.4)	70.4 (±8.0)	83.2 (±8.2)	100.8 (±13.7)	<0.001
Height (cm)	174.8 (±7.3)	175.6 (±7.6)	174.5 (±7.3)	174.2 (±6.8)	0.02
Waist (cm)	97 (±12)	87 (±7)	98 (±6)	112 (±10)	<0.001
Waist/hip	0.97 (±0.06)	0.93 (±0.06)	0.97 (±0.05)	1.00 (±0.05)	<0.001
Waist/height	0.56 (±0.07)	0.50 (±0.04)	0.56 (±0.04)	0.64 (±0.06)	<0.001
Smoker	232 (15.8%)	100 (21.0%)	96 (14.0%)	36 (11.8%)	0.001
Alcohol*(g/d)	12 (2–28)	12 (2–27)	13 (2–29)	11 (2–33)	0.7
EI*(MJ/d)	8.9 (7.2–11.1)	9.3 (7.3–11.3)	8.8 (7.1–11.0)	8.6 (6.9–11.1)	0.08
EI/BMR*	1.3 (1.0–1.6)	1.4 (1.1–1.7)	1.3 (1.0–1.6)	1.1 (0.9–1.4)	<0.001
EI/BMR<0.9*	154 (11.1%)	24 (5.4%)	67 (10.3%)	63 (21.6%)	<0.001

*n = 1388 (79 missing data).

BMI = body mass index (kg/m^2^); SD = standard deviation; IQR = interquartile range; EI energy intake (MJ/d); BMR = basal metabolic rate (MJ/d).

**Table 3:** Characteristics of women, overall and according to BMI categories; data presented as mean (±SD), median (IQR) or frequency (%).

	All	BMI category			
		<25.0	25.0–29.9	≥30	p
	n = 1076	n = 427	n = 333	n = 316	
Age (yr)	51 (35–66)	44 (30–63)	55 (41–70)	55 (41–66)	<0.001
Weight (kg)	72.6 (±16.1)	59.7 (±6.7)	71.4 (±6.9)	91.3 (±14.1)	<0.001
Height (cm)	162.2 (±6.9)	163.3 (±6.5)	162.0 (±7.2)	161.0 (±6.7)	<0.001
Waist (cm)	88 (±14)	76 (±8)	88 (±7)	104 (±11)	<0.001
Waist/hip	0.84 (±0.07)	0.81 (±0.07)	0.85 (±0.07)	0.87 (±0.06)	<0.001
Waist/height	0.54 (±0.09)	0.47 (±0.05)	0.54 (±0.05)	0.65 (±0.07)	<0.001
Smoker	151 (14.0%)	63 (14.8%)	41 (12.3%)	47 (14.9%)	0.5
Alcohol*(g/d)	3 (0–12)	5 (0–15)	3 (0–11)	1 (0–7)	<0.001
EI* (MJ/d)	6.6 (5.2–8.1)	6.7 (5.2–8.1)	6.6 (5.4–8.0)	6.6 (5.4–8.2)	0.9
EI/BMR*	1.1 (0.9–1.4)	1.2 (1.0–1.5)	1.1 (0.9–1.4)	1.0 (0.8–1.2)	<0.001
EI/BMR<0.9*	214 (20.6%)	63 (15.2%)	62 (19.0%)	89 (29.6%)	<0.001

*n = 1041 (35 missing data).

BMI = body mass index (kg/m^2^); SD = standard deviation; IQR = interquartile range; EI energy intake (MJ/d); BMR = basal metabolic rate (MJ/d).

Data from dietary analyses suggest no consistency in the pattern of total energy intake across BMI categories; however, there was an increase in the proportion of individuals with low EI/BMR (<0.9) with increasing BMI. No association was observed between self-reported alcohol intake and BMI for men, but among women, self-reported alcohol intake decreased with increasing BMI. A smaller proportion of obese men were smokers compared to those who were overweight or for whom BMI <25.0 kg/m^2^, but this pattern was not evident among women.

**Figure 1:**
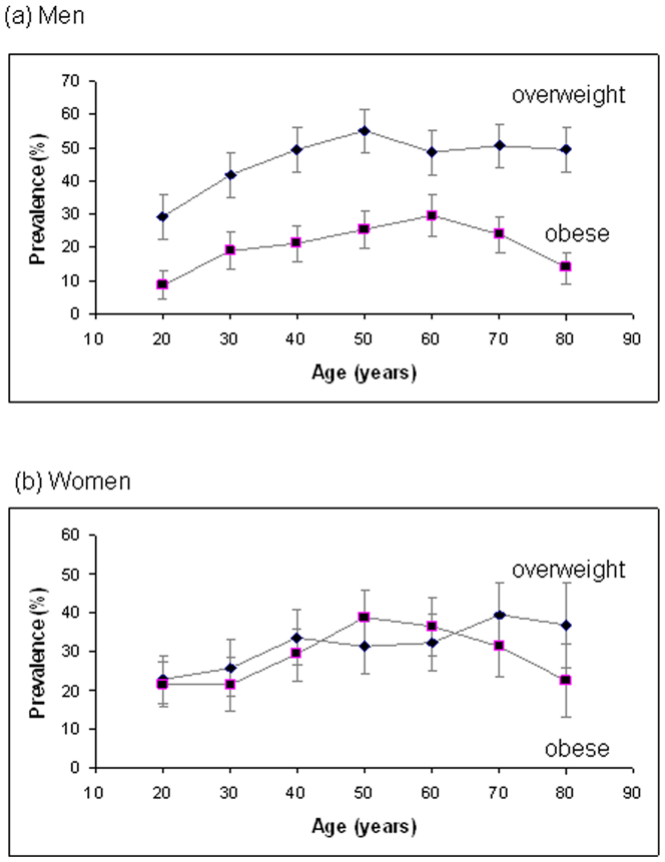
Age-specific prevalence of overweight and obesity. Age-specific prevalence (%) of overweight (body mass index, BMI, 25.0–29.9 kg/m^2^) and obesity (BMI ≥30.0 kg/m^2^) for (a) men and (b) women by age decades (20 = 20–29 years, etc). Data are shown as mean and 95% confidence intervals.

Age-specific prevalence of overweight and obesity as defined by BMI criteria are shown for men and women in Figure 1. Relatively low prevalence of obesity was observed for young men and women aged 20–29 years, and among the elderly aged ≥80 years; peaks of 29.7% (95%CI 23.5–35.9) occurred for ages 60–69 years for men and 38.7% (95%CI 31.7–45.7) for women aged 50–59 years. In contrast to women, the prevalence of overweight men exceeded the prevalence of obesity across the full adult age range.

### BMI compared with waist circumference for determining prevalence of obesity

According to waist circumference, our data indicate that 25.4% of individuals were overweight and a further 36.9% were obese; prevalence figures for each gender are listed in Table 1. The prevalence of obesity based on waist circumference was lower for men than for women (RR = 0.69, 95%CI 0.63–0.76). Pooled overweight and obese data indicate that 56.8% men and 67.5% women had waist measurements that exceeded the recommended threshold (62.3% overall).

There was exact agreement using BMI and waist circumference criteria for categorising normal, overweight and obese groups for 66.1% men and 67.8% women; agreement to within one category was observed for 99.5% men and 96.4% women. Obesity defined by waist circumference identified a larger proportion of the population than did obesity defined by BMI. Whereas 95.3% of the women and 90.8% of the men who were classed as obese by BMI were also obese by waist circumference, only 60.4% women and 55.5% men who were classed as obese using waist circumference were also obese using BMI.

### BMI and body fat

**Figure 2:**
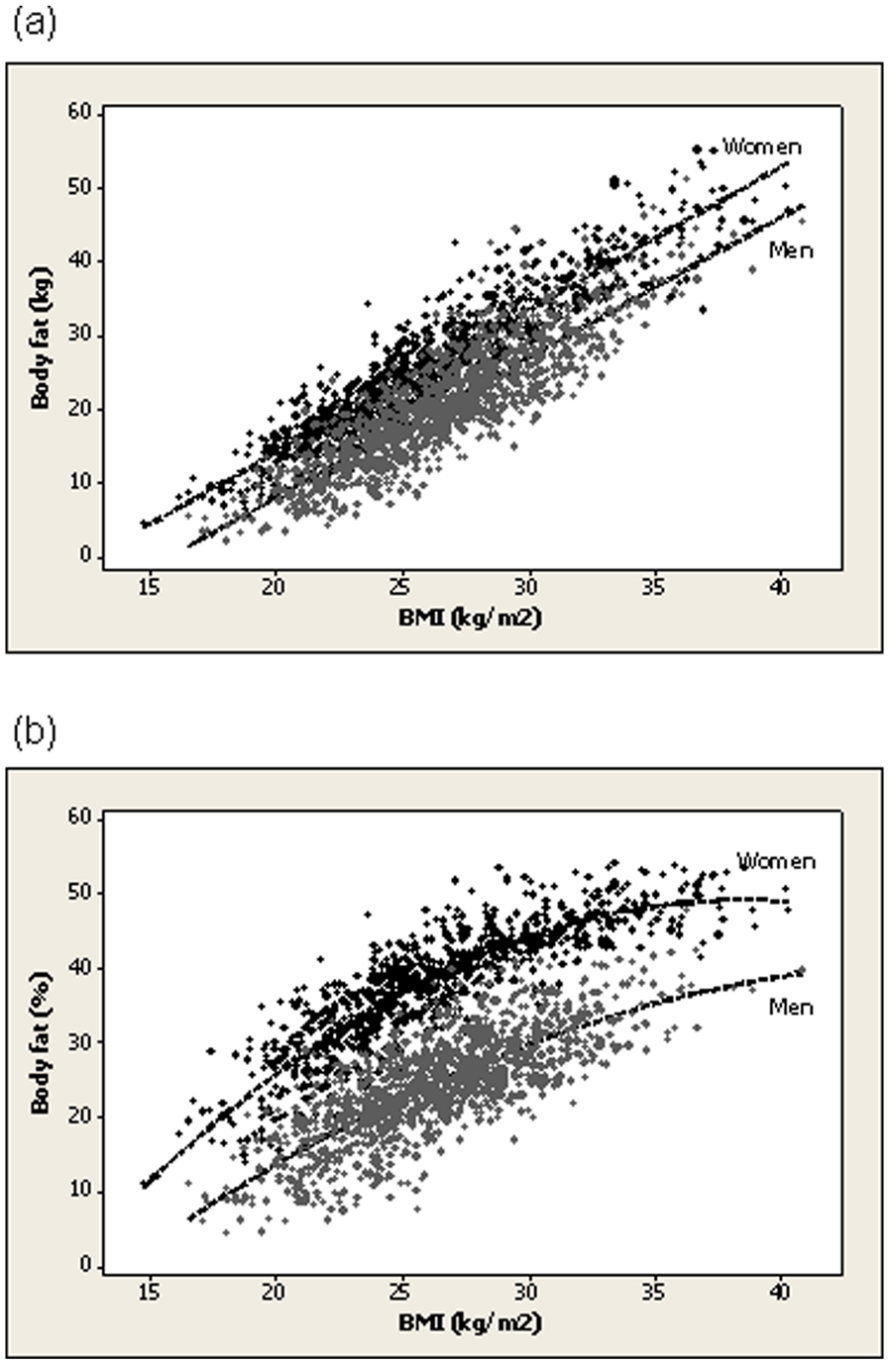
Body mass index and the relationship with body fat. Scatter plot of body mass index (BMI) against (a) body fat (kg) for men and women and (b) body fat (%). Predicted values are represented by dashed lines.

A linear relationship was observed between BMI and body fat mass (Figure 2a). For any given BMI in the range, mean body fat mass was 6.3 kg greater for women than for men (p<0.001). For BMI 25.0 kg/m^2^, the mean predicted body fat mass was 17.7 kg (95%CI 17.5–17.9) for men and 24.0 kg (95%CI 23.7–24.3) for women; for BMI 30.0 kg/m^2^, the mean predicted body fat mass was 27.2 kg (95%CI 27.0–27.5) for men and 33.5 kg (95%CI 33.2–33.8) for women.

Gender was identified as an effect modifier in the second order polynomial relationship between BMI and %BF (Figure 2b). Over the range of BMI, for any particular BMI, %BF was greater for women than for men. For BMI 25.0 kg/m^2^, the mean predicted %BF was 22.7% (95%CI 22.4–23.0) for men and 36.7% (95%CI 36.4–37.0) for women; for BMI 30.0 kg/m^2^, the mean predicted %BF was 29.9% (95%CI 29.6–30.2) for men and 44.2% (95%CI 43.8–44.6) for women.

Table 4 lists regression coefficients for regression models for predicting the dependent variable, %BF, by sequentially including the independent variables gender, BMI and age. The prediction equation combining these variables explained 0.825 of the variance in %BF. When lean mass and BMC were also included as independent variables in the models, the contribution of age was no longer significant (p>0.05). The regression coefficients for the parsimonious model for predicting %BF are shown in model 5 (Table 4); the model includes gender, BMI, lean mass and BMC as the independent variables, which explains 0.889 of the variance in %BF. Gender was identified as an effect modifier, so interaction terms are also included.

**Table 4:** Regression coefficients (95%CI) for models predicting the dependent variable, %BF, by sequentially including gender (model 1), BMI (model 2), age (model 3), lean mass (model 4) and BMC (model 5) as independent variables.

Model	Independent variables	Coefficient (95%CI)	p	R^2^	S
1	gender	−13.1 (−13.7 to −12.5)	<0.001	0.433	7.33
	constant	37.6 (37.1 to 38.1)	<0.001		
2	gender	−14.2 (−14.7 to −13.8)	<0.001	0.813	4.21
	BMI-mean	1.65 (1.58 to 1.72)	<0.001		
	(BMI-mean)^2^	−0.06 (−0.07 to −0.05)	<0.001		
	gender*(BMI-mean)	−0.14 (−0.23 to −0.04)	0.004		
	gender*(BMI-mean)^2^	0.03 (0.01 to 0.04)	0.001		
	constant	39.1 (38.7 to 39.4)	<0.001		
3	gender	−16.7 (−17.8 to −15.6)	<0.001	0.825	4.07
	BMI-mean	1.62 (1.56 to 1.69)	<0.001		
	(BMI-mean)^2^	−0.06 (−0.07 to −0.05)	<0.001		
	age	0.02 (0.01 to 0.04)	0.001		
	gender*(BMI-mean)	−0.17 (−0.26 to −0.08)	<0.001		
	gender*(BMI-mean)^2^	0.03 (0.01 to 0.04)	<0.001		
	gender*age	0.04 (0.03 to 0.06)	<0.001		
	constant	37.8 (37.0 to 38.7)	<0.001		
4	gender	−9.8 (−12.6 to −7.09)	<0.001	0.884	3.31
	BMI-mean	1.84 (1.78 to 1.89)	<0.001		
	(BMI-mean)^2^	−0.06 (−0.07 to −0.05)	<0.001		
	lean mass	−0.55 (−0.61 to −0.50)	<0.001		
	gender*(BMI-mean)	0.09 (0.01 to 0.17)	0.032		
	gender*(BMI-mean)^2^	0.02 (0.00 to 0.03)	0.016		
	gender*lean mass	0.10 (−0.04 to 0.16)	0.001		
	constant	60.8 (58.6 to 63.0)	<0.001		
5	gender	−10.2 (−12.9 to −7.5)	<0.001	0.889	3.24
	BMI-mean	1.78 (1.73 to 1.84)	<0.001		
	(BMI-mean)^2^	−0.05 (−0.06 to −0.04)	<0.001		
	lean mass	−0.73 (−0.80 to −0.66)	<0.001		
	BMC	2.79 (2.11 to 3.47)	<0.001		
	gender*(BMI-mean)	0.15 (0.07 to 0.23)	<0.001		
	gender*(BMI-mean)^2^	0.02 (0.00 to 0.03)	0.018		
	gender*lean mass	0.22 (0.14 to 0.29)	<0.001		
	gender*BMC	−1.42 (−2.28 to −0.56)	0.001		
	constant	60.3 (58.2 to 62.4)	<0.001		

CI = confidence interval; %BF = percentage body fat; gender: men = 1, women = 0; BMI = body mass index (kg/m^2^); age (yr); mean BMI = 26.37 kg/m^2^; lean mass (kg); BMC = bone mineral content (kg); S = standard error of the regression.

Polynomial relationships were centred about the mean to reduce collinearity. Interaction terms are also included.

## Discussion

According to internationally accepted thresholds for BMI, 37.5% of individuals (45.1% of men and 30.2% of women) were overweight and a further 24.5% were obese (20.2% of men and 28.6% of women). Prevalence figures using waist circumference were 25.4% overweight (27.5% of men and 23.3% of women) and 36.9% obese (29.3% of men and 44.1% of women). Both criteria indicated that approximately 60% of the population exceeded the recommended threshold for healthy body habitus. An increase in the proportion of men and women with low EI/BMR was observed with increasing BMI and this could reflect deliberate caloric restriction practised by overweight and obese individuals and/or underreporting of dietary intake that is accentuated with greater BMI [22], [23].

Our estimated 24.5% (95%CI 23.4–27.2) prevalence of obesity for the period 2001–2008 suggests an increase since 1999–2000, when the nationwide AusDiab study reported a prevalence of 20.8% (95%CI 18.4–23.1) [11]. This comparison is tempered, however, by differences in sampling strategies that exist between the studies. In contrast to the AusDiab study, we observed a greater prevalence of obesity in women than in men for estimates based on both BMI and waist circumference. Consistent with that earlier report, and also based on cross-sectional data, we have documented that the prevalence of obesity increased with age until after middle age and declined in old age. This decline may be due partly to obesity-related mortality [11].

Determining BMI does not require sophisticated equipment and it is easy to calculate; however, we have highlighted limitations in its use as a measure of adiposity. Gender-specific differences in the relationship between BMI and body fat were largely explained by the greater lean mass associated with a muscular male body build and bone mass. Excess weight-for-height attributable to lean and bone tissue rather than body fat may, in part, account for the observed high prevalence of overweight men according to BMI criteria. We also report an effect of age on %BF that was independent of BMI. Thus, there is a likely underestimation of adiposity by BMI in elderly people for whom there is a loss of lean tissue, particularly skeletal muscle, and bone. An age-related decline in DXA lean mass, particularly in men, has been observed in apparently healthy individuals, in the absence of accompanying weight loss [24]. The observed age-related changes in body composition are consistent with reported effects of growth hormone deficiency and the increasing prevalence of growth hormone deficiency with age [25] together with an age-related decline in sex steroids, particularly testosterone in men [26].

Our reported non-linear relationship between BMI and %BF, but linear relationship between BMI and body fat mass, underscores an earlier observation by Garrow and Webster that the correlation between of BMI with %BF is not as strong as the correlation with body fat mass [27]. Studies in children and adolescents [28], [29] have similarly reported that BMI is more poorly correlated with DXA estimates of %BF than with body fat mass. BMI is indicative of body fat mass not the relative measure expressed as a percentage of body weight; increments in body weight result in diminishing increments in %BF.

We recognise several strengths and weaknesses in our study. The age-stratified random sampling technique used to generate our sample is a strength that ensured a good representation of all ages across the adult age spectrum. We acknowledge that prevalence data for obesity may be influenced by incomplete participation in the study and a bias related to body composition cannot be excluded; no body composition data were available for non-participants. The use of a bone densitometer for measuring body fat, lean and bone tissue precludes large individuals as the scanner bed has a safety load limit and the bed dimensions do not accommodate very large bodies. Thus, relationships between BMI and body fat have not included the very obese. Similarly, a frailty bias may have been introduced by excluding individuals with prostheses or pacemaker implants. The sample was essentially white and, as the relationship between body fat and BMI differs by ethnicity [30], our findings may not be generalisable to other populations. Different thresholds for overweight and obesity defined by anthropometry have been developed to cater for recognised ethnic differences [12].

In conclusion, we report a high prevalence of overweight and obese men and women in Australia, based on BMI and waist circumference criteria. In the absence of sophisticated imaging technologies such as DXA in most clinical settings, body fat is difficult to measure, supporting a role for using BMI to estimate health risk. However, we have demonstrated that excess body weight-for-height may not necessarily be indicative of excess body fat. Our data support an earlier contention that, to improve estimates of excess body fat, BMI thresholds for defining overweight and obesity should be gender- and age-specific to account for differences in body build in men and women and the effects of age on body composition [31], [32]. Adiposity is a continuous trait and we recognise that optimal thresholds will remain dependent on risk assessment for morbidity and mortality.
